# Atractylone in the *Atractylodes macrocephala* Rhizoma Essential Oil and Its Anti-Inflammatory Activity

**DOI:** 10.3390/molecules28217340

**Published:** 2023-10-30

**Authors:** Ling Li, Yihao He, Nan Wang, Yuting Li, Yaoyao Du, Ning He, Bing Wang, Tong Zhang

**Affiliations:** 1 School of Pharmacy, Shanghai University of Traditional Chinese Medicine, Shanghai 201203, China; liling_sh@163.com (L.L.); yhhe5668@163.com (Y.H.); w_nan8797@163.com (N.W.); yydu_lk1130@163.com (Y.D.); 2 College of Pharmacy, Anhui University of Chinese Medicine, Hefei 230012, China; hening@ahtcm.edu.cn; 3 Shanghai Institute of Materia Medica, Chinese Academy of Sciences, Shanghai 201203, China; 4 Department of Dermatology, Yueyang Hospital of Integrated Traditional Chinese and Western Medicine, Affiliated to Shanghai University of Traditional Chinese Medicine, Shanghai 200437, China; 22023329@shutcm.edu.cn

**Keywords:** *Atractylodes macrocephala* rhizoma essential oil, atractylone, signaling pathway, anti-inflammation, antioxidative stress, intestinal injury reduction

## Abstract

The aim of this study was to conduct a screening of potential therapeutic compounds found in the *Atractylodes macrocephala* rhizoma essential oil (AO) and explore its mechanism of action in the treatment of ulcerative colitis (UC). An inflammation cell model was employed in conjunction with phospho-antibody array technology to explore potential therapeutic compounds of AO and their anti-inflammatory and antioxidant effects. Furthermore, we assessed their efficacy and mechanisms of action in treating dextran sulfate sodium (DSS)-induced colitis in mice. Via the screening process, we identified atractylone (ATR) as the primary active compound in AO. It has been demonstrated that ATR can both decrease the levels of tumor necrosis factor (TNF)-α and reactive oxygen species (ROS) and increase the expression of adhesion proteins such as claudin, ZO-1, and occludin in vitro. Moreover, ATR has been shown to improve UC symptoms in vivo. Via a non-targeted metabolomics analysis of colon tissue, we identified 57 distinct metabolites that responded to ATR treatment. Subsequent analysis of the metabolic pathways revealed that the action of ATR was primarily focused on the amino acid metabolism pathway. In summary, ATR may alleviate the symptoms of UC by regulating multiple signaling pathways. Additionally, ATR has a comprehensive function in anti-inflammation, antioxidative stress, and intestinal injury reduction.

## 1. Introduction

Ulcerative colitis (UC) is a chronic inflammatory disease of the colon and rectum [1], characterized by a course of relapsing and remitting symptoms. The defining features of UC encompass bloody diarrhea, along with the occurrence of rectal urgency and tenesmus [2]. The selection of treatment approaches is contingent upon the extent of the disease, with aminosalicylates typically being the preferred choice for mild–moderate cases of UC. Topical and systemic steroids can be used to treat UC recurrences, while immunosuppressants and biological drugs are used in moderate–severe cases. However, these interventions exhibit a restricted therapeutic impact, often accompanied by a low rate of response [3,4]. The utilization of biological treatments introduces substantial challenges, particularly in the field of biologics, which has a profound influence on patients. Nonetheless, traditional Chinese medicine provides a fresh approach to the treatment of UC due to its broad pharmacological activities, multiple targets, and minimal side effects.

*Atractylodes macrocephala* rhizoma (AMR), the dried rhizomes of *Atractylodes macrocephala* Koidz., has been used for thousands of years to treat a broad spectrum of diseases, including spleen hypofunction, loss of appetite, abdominal distension, and diarrhea, which are typical manifestations of UC [5,6]. An animal study also showed that AMR extracts surpassed sulfasalazine in their ability to prevent dextran sulfate sodium (DSS)-induced colitis. These extracts from AMR could potentially serve as natural agents to prevent IBD relapse [7]. Moreover, treatments involving AMR’s essential oil (AO) exhibited significant suppression of nitric oxide (NO) and prostaglandin E2 (PGE2) production (synthesis) in lipopolysaccharide (LPS)-stimulated RAW264.7 cells, highlighting the anti-inflammatory activity of AO [8]. In addition, a previous study has indicated that an imbalance in mucosal immunity, stemming from the apoptosis of colonic epithelial cells, can expedite the evolution of UC [9]. Nevertheless, the efficacy and mechanism of action of ATR in restoring the intestinal epithelial barrier, in combination with providing anti-inflammatory relief for colitis, remain unexplored.

In this study, the signaling pathway was screened with antibody chip technology. Using a patented three-dimensional polymer membrane (PEX100), we successfully immobilized 1318 high-specificity antibodies with high density, allowing the detection of phosphorylation at 679 sites on 432 signal proteins. To improve the sensitivity and stability of phosphorylation detection, the phosphorylation site statuses of individual specific proteins were probed employing a pair of antibodies consisting of one designed for a phosphorylated (phospho) state and another intended for a non-phosphorylated (nonphospho) state.

Based on our analysis of its anti-inflammatory and antioxidant properties, ATR has been identified as the main active compound in AO. Additionally, we established an in vitro inflammation cell model to assess the anti-inflammatory effects of ATR at various concentrations and to evaluate its capacity to restore intestinal barrier function. Subsequently, we validated the therapeutic efficacy of ATR by identifying relevant pathways using antibody arrays and conducting experiments on a UC mouse model. Additionally, we conducted metabolomics analysis to evaluate the impact of ATR on the modulation of amino acid metabolism in mitigating colonic inflammation.

## 2. Results

### 2.1. Atractylone Is a Pivotal Pharmacologically Active Compound

**Figure 1:**
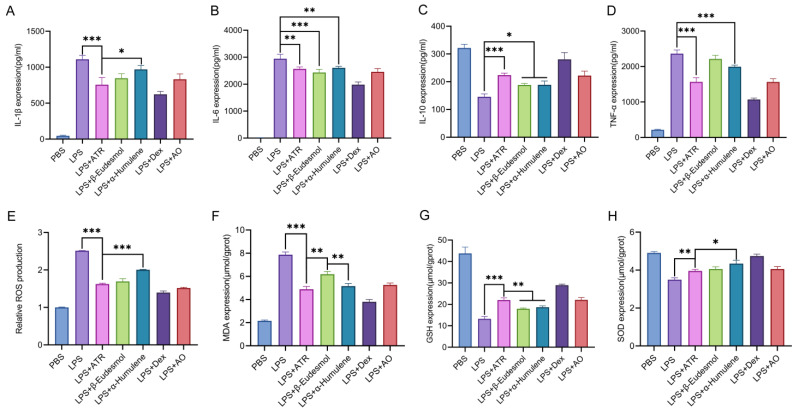
Anti-inflammatory and anti-oxidation effects of ATR, β-eudesmol, α-humulene, dexamethasone, and AO in vitro. (**A**–**D**) RAW 264.7-cell-secreted inflammatory cytokines were examined using ELISA. (**E**) Statistical analysis of ROS levels. The levels of (**F**) MDA, (**G**) GSH, and (**H**) SOD were measured. Data are expressed as means ± SD (n = 3). * *p* < 0.05, ** *p* < 0.01, and *** *p* < 0.001.

The contents of AO components and the in vitro anti-inflammatory and antioxidant activities of each component in the pre-test were considered comprehensively. This study examined the anti-inflammatory and antioxidant effects of three components (ATR, β-eudesmol, and α-humulene) found in AO when tested in RAW264.7 cells. We assessed the changes in inflammatory factors, as well as SOD, MDA, and GSH levels, following cell treatment. Furthermore, we evaluated the variations in ROS levels using flow cytometry. As shown in Figure 1, at equipotent concentrations, ATR, β-eudesmol, and α-humulene exhibited substantial anti-inflammatory and antioxidant effects. ATR constituted the highest content in AO [10]. Based on this observation, it is speculated that ATR may function as the primary active ingredient responsible for the efficacy of AO.

### 2.2. ATR Inhibits LPS-Induced Inflammatory Reactions in RAW264.7 Cells

**Figure 2:**
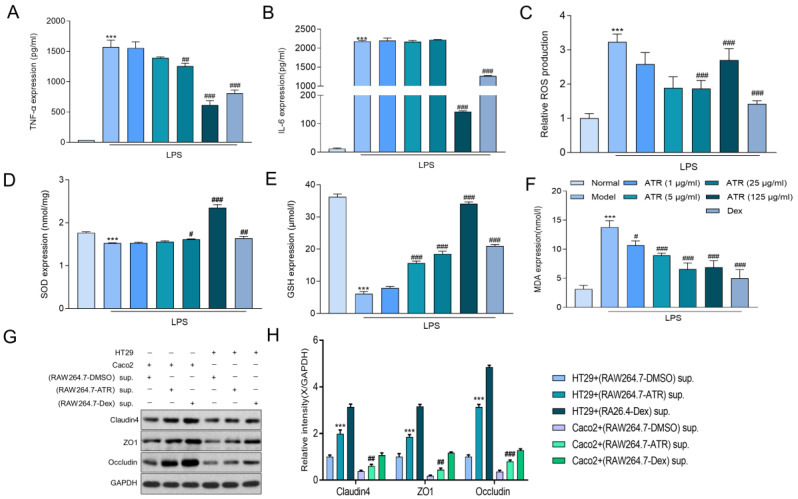
Effects of ATR on inflammatory mediator expression in LPS-treated RAW 264.7 cells. (**A**,**B**) RAW 264.7-cell-secreted proinflammatory cytokines were examined using ELISA. (**C**) Statistical analysis of ROS levels. The levels of (**D**) SOD, (**E**) GSH, and (**F**) MDA were measured. (**G**,**H**) The expression of claudin/ZO-1/occludin in HT29/Caco2 cells was detected via Western blotting using supernatant from RAW264.7 cells treated with ATR. Data are expressed as means ± SD (n = 3). *** *p* < 0.001 vs. the normal group. # *p* < 0.05, ## *p* < 0.01, and ### *p* < 0.001 vs. the model group.

To explore the anti-inflammatory effect of ATR on RAW264.7 cells, alterations in TNF-α, IL-6, SOD, MDA, and GSH were examined using ELISA, and ROS were examined using flow cytometry. The results depicted in Figure 2A,B demonstrate that ATR at various concentrations (1, 5, 25, and 125 μg/mL) significantly inhibited the secretion of TNF-α in LPS-induced RAW264.7 inflammatory cells (*p* < 0.05). However, only a high dosage (125 g/mL) of ATR was able to suppress the proinflammatory cytokine IL-6 in a dose-independent manner. It also suppressed the secretion of ROS (Figure 2C). In addition, the levels of antioxidant enzymes SOD and GSH exhibited a substantial increase at different concentrations of ATR compared with the control group (*p* < 0.001). ATR pre-administration led to an increase in antioxidant enzymes in a dose-dependent manner (Figure 2D,E). Simultaneously, when compared with the LPS-treatment group, the MDA level decreased in a dose-dependent manner after adding different concentrations of ATR, with a statistically significant difference (*p* < 0.001) (Figure 2F).

### 2.3. ATR Is Effective in Restoring the Intestinal Mucosal Barrier

HT29 and Caco2 cells were cultured in the supernatant of RAW264.7 cells after drug treatment to evaluate the effect of ATR on the intestinal mucosal barrier. Subsequently, the expression levels of the tight junction proteins ZO-1, occludin, and claudin 4 were measured (Figure 2G,H). According to the results, the tight junction protein concentration in the ATR group was considerably greater than that in the control group. After treatment with ATR and Dex, the tight junction protein concentration increased in both HT29 and Caco2 cells, with similar impacts observed in both cell types.

### 2.4. Phosphorylation Antibody Chip Analysis

**Figure 3:**
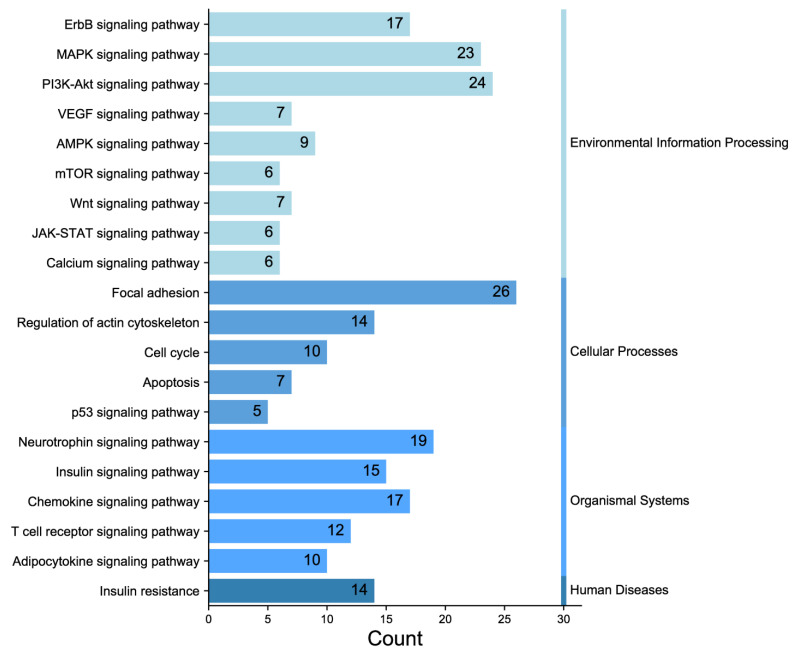
Classification diagram of pathway enrichment results.

**Figure 4:**
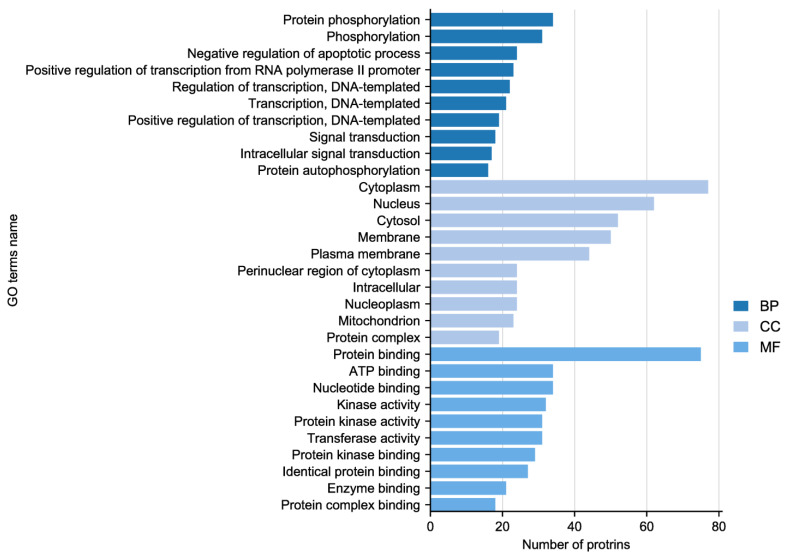
Gene Ontology (GO) enrichment analysis of altered sites showing enriched GO terms.

In the experiment, the quality of two sample images and the data met the established criteria. Subsequently, 584 paired phosphate sites were compared to figure out the differences between samples. KEGG serves as the main database for the systematic analysis of gene function, genome, and proteome information. Different proteins coordinate with each other to perform their biological functions, which helps researchers study proteins and their expression information as a whole network. As illustrated in Figure 3, the KEGG pathways identified the presence of focal adhesion, primarily involving the PI3K–AKT signaling pathway and the MAPK signaling pathway. GO enrichment analysis revealed that proteins with significantly altered phosphorylation statuses were highly enriched in processes such as protein phosphorylation, phosphorylation, and the negative regulation of the apoptotic process, and cellular locations like the cytoplasm, nucleus, cytosol, as well as protein binding, ATP binding, and nucleotide binding (Figure 4).

### 2.5. Experimental Validation of ATR’s Impact on UC In Vivo

**Figure 5:**
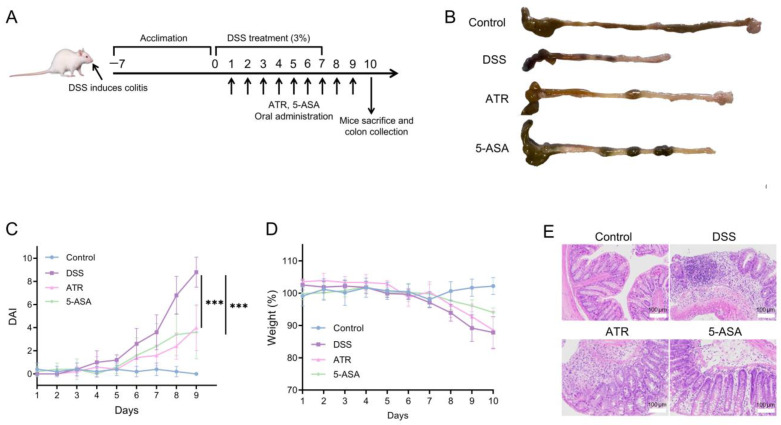
Experimental validation of AO’s impact on UC in vivo. (**A**) Schematic diagram of colitis induction and treatment. (**B**) Morphology of the colon in mice. Changes in DAI (**C**) and weight (**D**) during the treatment. (**E**) H&E-stained histological sections of colonic tissues. n = 5, *** *p* < 0.001.

The treatment study was carried out in DSS-induced colitis mice (Figure 5A). A 7-day dose of 3% DSS in mice elicited typical colitis symptoms, including weight loss, colon shortening, and hematochezia. The body weight of mice in the DSS group did not drop until day 7. After drug treatment, there was a drop in the DAI (Figure 5C), and the UC symptoms of DSS-treated mice exhibited a remarkable improvement. The weight of the ATR group increased during therapy compared with that of the DSS group (Figure 5D). Colon length serves as a crucial indicator of colitis conditions. The colons in the DSS group were shortened in comparison with those in the control group, while the colons in the treatment groups were longer than those in the DSS group (Figure 5B), indicating improvement. ATR exhibited a comparable impact on UC treatment to that of the positive control medication 5-ASA.

According to a prior study [10], the primary component of AO is ATR (41.92%). Both AO and ATR can greatly reduce the release of NO in ANA-1 cells produced by LPS, with ATR demonstrating remarkably higher anti-inflammatory efficacy than essential oils in vitro. In this research, the dose of ATR used in the animal studies was 50 mg/kg. When compared with 5-ASA, ATR has a preferable therapeutic effect in reducing weight loss, DAI scores, and colon shortening (Figure 5). Moreover, analysis of the H&E-stained sections unveiled extensive damage to the colonic structure due to DSS-induced colitis, characterized by a disrupted epithelium, depleted goblet cells, and the infiltration of inflammatory cells. ATR treatment ameliorated inflammation in the colon tissue of mice, leading to the restoration of mucosal structure and a reduction in the infiltration of inflammatory cells (Figure 5E).

### 2.6. Analysis of Potential Metabolites in Colon Tissue

**Figure 6:**
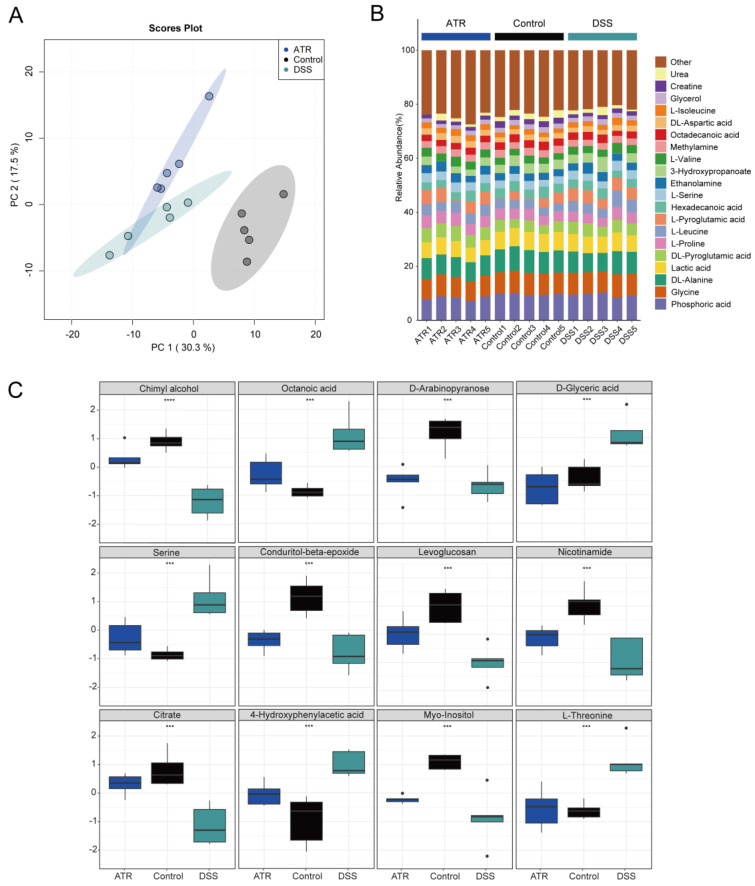
The effect of ATR on endogenous metabolites in colon tissue of mice with ulcerative colitis. (**A**) Stacked bar chart of percentage compositions of the top 20 metabolites by abundance. (**B**) PCA cloud image. (**C**) Box plots of differential metabolites. n = 5, *** *p* < 0.001 and **** *p* < 0.0001.

Metabolite detection in mouse colon tissue was carried out using GC-TOF-MS, and data analysis was conducted using the R package MetaboAnalyst 4.0. Unsupervised PCA modeling was employed to investigate the data, as depicted in Figure 6A. The distribution of point clouds for each group was observed to be significantly different, indicating significant variations in the metabolic compositions of the colon tissues between groups. Furthermore, for comprehensive comparison, the percentage of each metabolite in the samples was calculated and is visualized in Figure 6B. Additionally, notable differences in metabolite levels between groups were examined, as presented in Figure 6C. In particular, the levels of octanoic acid, serine, D-glyceric acid, 4-hydroxyphenylacetic acid, and L-threonine were found to be significantly higher in the DSS group compared with the normal group. Conversely, chimyl alcohol, D-arabinopyranose, conduritol-beta-epoxide, citrate, myo-inositol, levoglucosan, and nicotinamide exhibited decreased levels in the DSS group. Interestingly, after drug treatment, the levels of these metabolites showed an opposite trend, indicating that drug treatment may have a significant impact on the metabolic changes observed in UC mouse colon tissue. Additional research is needed to ascertain the specific mechanisms underlying the effects of drug treatment on these metabolites and their potential therapeutic benefits in UC. Overall, these findings highlight the importance of utilizing metabolomics approaches to identify and characterize differences in metabolite profiles associated with various diseases, such as UC. This information can be conducive to the identification of potential biomarkers and therapeutic targets for disease diagnosis and treatment. Finally, according to the integrated pathway analysis, multiple pathways were found to be affected. These pathways ranged from amino acid metabolism, such as arginine and proline metabolism, alanine, aspartate, and glutamate metabolism, to purine metabolism, including aminoacyl-tRNA biosynthesis (Supplementary Figure S1).

## 3. Discussion

UC is characterized by recurrent bouts of inflammation and a relatively protracted illness course. It involves immunological abnormalities in the gastrointestinal tract that are induced by chronic intestinal inflammation. The disease tends to have a protracted course with frequent recurrences, posing challenges for effective therapy. Consequently, the development of a safe and effective therapy remains a significant issue [11]. Currently, due to its multi-target mechanism and fewer side effects, traditional Chinese medicine may be able to provide distinct benefits in the treatment of chronic diseases, including an accurate curative impact, low toxicity, and effectiveness. In the treatment of UC, Chinese medicine also showcases noteworthy qualities [12].

Preliminary studies and animal experiments demonstrate that ATR has notable potential in the treatment of UC. KEGG signaling pathway enrichment analyses provide support for the notion that the signaling pathways implicated in the core target of ATR are mainly the MAPK and PI3K–AKT signaling pathways. The conventional, immune–inflammatory signaling pathway is the most significant. Simultaneously, the intestinal barrier has been shown to play an important role in preserving intestinal health [13]. The etiology of UC involves the frequent synergistic activation of several pathways, resulting in the release of numerous proinflammatory agents and promoting mucosal epithelial healing following intestinal injury. The expression levels of critical pathway proteins, including p-PI3K and p-AKT, were observed to be elevated in animal models of UC, colon cancer, and colon cancer associated with colitis [14,15,16]. Cell proliferation, differentiation, migration, and death are all regulated via both MAPK signaling pathways and conserved signaling pathways [17].

It is possible that ATR can alleviate UC symptoms by lowering the levels of the inflammatory factor TNF-α and inhibiting the activation of the PI3K–AKT signaling pathway. We conducted a comprehensive, pharmacology-based study on the phosphorylation of the protein chip to elucidate the possible active components of AO and the underlying processes contributing to its positive effects on UC. The upregulation of IL-6 and TNF-α mRNA expression has been linked to the pathophysiology of UC in several studies [18,19]. It is worth mentioning that the therapeutic use of anti-TNF-α drugs has been utilized to treat UC [20,21]. TNF plays diverse proinflammatory roles in colitis by binding to the TNFR1 and TNFR2 receptors and activating the PI3K–AKT pathway within cells [22]. In vitro studies have confirmed that ATR can mitigate the generation of inflammatory factors such as IL-6 and TNF-α, thereby effectively counteracting LPS-induced inflammatory symptoms in RAW264.7 cells.

Based on gene ontology and inferences from the literature, ATR appears to modulate oxidative stress in colonic tissue by increasing SOD, decreasing ROS and MDA levels, as well as regulating the chemical stress response. ROS play a crucial role [23,24] in the pathophysiology of IBD. Scavenging ROS with SOD is one of the most successful ways of preventing IBD, which implicates balancing pro-oxidation and antioxidation to generate an antioxidative impact. This approach has shown promising results in treating experimental colitis [25]. By scavenging oxygen-free radicals and lowering lipid peroxides, SOD can reduce the intestinal inflammatory response. In vitro investigations have indicated that ATR could reduce the production of ROS and MDA in RAW264.7 cells while also promoting the secretion of SOD and GSH in a dose-dependent manner.

By modulating MAPK signaling, ATR encourages tight proteins to restore the intestinal barrier. The MAPK signaling system, including ERK1/ERK2, JNK/SAPK, and p38 MAPK, is implicated in intestinal mucosal injury in inflammatory bowel disease (IBD) [26]. Previous research has found that structural abnormalities in TJ proteins, such as decreased ZO-1 and occludin, cause increased intestinal permeability in IBD patients [27]. Although ZO-1 deletion in intestinal epithelial cells does not affect the function of the intestinal barrier, it can lead to deficiencies in repairing mucosal damage by inhibiting intestinal epithelial cell proliferation [28]. In our study, ATR strongly prevented the LPS-induced loss of ZO-1, claudin 4, and occludin levels in HT29 and Caco2 cells, implying that ATR may protect barrier integrity by preserving the expression of ZO-1, claudin 4, and occludin. This preservation could potentially alleviate the severity of colitis.

The metabolomics results showed that there were seven downregulated and five upregulated metabolites in the differential metabolites in UC mouse colon tissue, which exhibited an opposite trend after drug treatment. There is significant evidence suggesting that dietary inositol, along with its phosphates and phospholipid derivatives, plays a beneficial role in human health. The experimental results show that the content of inositol decreased in differential metabolites after DSS modeling. However, mice treated with inositol significantly inhibited the activity index of ulcerative colitis, especially in terms of ulcer formation and inflammatory area [29]. Moreover, treatment with myoinositol has been found to block the activation of AKT and β-catenin in the colitis model induced by DSS [30]. It is speculated that the regulation of inositol content in tissues after ATR treatment plays a role in the anti-colitis effect. Dadi Xie et al. discovered that the amino acid metabolism profiles of rats treated with DSS were significantly different from those of the control group. This finding suggests a potential correlation between changes in amino acids and the pathogenesis of colitis. Furthermore, specific amino acids may potentially serve as biomarkers for IBD patients [31]. Based on our experimental results, it appears that the therapeutic efficacy of ATR may be partially attributed to alterations in the metabolic pathways of amino acids [32]. Multiple studies have indicated that glutamine contributes to intestinal cell oxidative metabolism and the regulation of oxidative reactions. This is achieved via its role as a substrate for protein synthesis, providing energy substrates for tissue mucosa, synthesizing substrates for glutathione (GSH), and generating metabolites derived from glutamine [33,34,35]. A study conducted by Li et al. demonstrated that administering glutamine significantly boosts cell proliferation and antioxidant levels. Moreover, it reduces apoptosis in colonic mucosal cells, downregulates the expression of Bax and caspase-3, and decreases the levels of pro-inflammatory factors in the colonic mucosa [36]. These findings indicate that amino acids could potentially serve as a promising adjunctive treatment strategy for enhancing inflammation and preventing complications. Subsequently, there will be further investigation into the pathogenesis of UC and the identification of novel therapeutic targets.

ATR is extremely unstable at room temperature, leading to the rapid transformation of most of its pure products into atractylenolides I and III [37]. The stability of ATR in AO varies under different conditions. The relative content of ATR in room-temperature sunlight, room-temperature darkness, artificial gastric fluid, and artificial intestinal fluid is significantly decreased, while AO remains nearly insoluble in water. At room temperature, the decomposition rate of ATR reached more than 30% on the fifth day of storage under the condition of avoiding light, but at −20 °C, ATR basically did not decompose for five days [38]. As reported, the cyclodextrin inclusion approach can prevent volatile oil from being oxidated and breaking down, while it can also convert the liquid medicine into a powder to improve the drug’s stability during both preparation and storage operations [39]. Tang et al. developed stomach-targeted AO pellets for the synergistic treatment of UC [40]. In addition, Weiwei Shi et al. created a microemulsion of cinnamon essential oil to address its quick disintegration and oxidation vulnerabilities [41]. Subsequent studies will focus on developing a novel nano dosage form of ATR to increase its solubility and stability, thus amplifying its anti-UC effect.

## 4. Materials and Methods

### 4.1. Preparation of AO and ATR

ATR was purchased from Shanghai Hongyong Biotechnology Co., Ltd. (Shanghai, China). AO was extracted in our team’s laboratory, adopting a heavy volatile oil extraction device. The details are as follows: 200 g of AMR, which was smashed through a No. 2 sieve, was added to 1000 mL of water. After thorough shaking and mixing, the mixture was heated and maintained in a slightly boiling state for 5 h, or until the amount of oil collected in the device ceased to increase. It was then left to stand for over 1 h before the volatile oil was collected. Gas chromatography–mass spectrometry (GC-MS) was utilized to isolate volatile oil components from the AMR in previous work [10].

### 4.2. Effects of AO on Antioxidant Properties and Inflammatory Responses

Components containing more than 1% of AMR’s essential oil were incubated with RAW 264.7 macrophages to evaluate the anti-inflammatory and antioxidant properties of each component. ATR, β-eudesmol (200431-221101, 98.96%), and α-humulene (230381-221101, 94.87%) were purchased from Anhui Xiqingguo Biotechnology Co., Ltd. (Hefei, China). Dexamethasone (S132203) was purchased from Selleck Co., Ltd. (Houston, TX, USA), as well as the essential oil itself. Macrophages were subjected to stimulation with lipopolysaccharides at a concentration of 10 μg/mL for a duration of 12 h, and RAW 264.7 macrophages were exposed to different volatile oil components at a concentration of 25 μg/mL for a period of 8 h. Finally, the concentrations of inflammatory cytokines (TNF-α, IL-6, IL-1β, and IL-10) were quantified via ELISA. The cells were evaluated for superoxide dismutase (SOD), malondialdehyde (MDA), and glutathione (GSH) levels and total ROS via a commercial kit and flow analysis.

### 4.3. ATR Inhibits Inflammatory and Oxidative Stress In Vitro

#### 4.3.1. Cells

RAW264.7, HT29, and Caco2 cells were purchased from the American Type Culture Collection (Manassas, VA, USA). The cells were cultured in DMEM supplemented with 10% (*v*/*v*) fetal bovine serum and 1% penicillin–streptomycin antibiotics. The cells were maintained in a humidified incubator set at a temperature of 37 °C and supplemented with 5% CO_2_.

#### 4.3.2. TNF-α and IL-6 Levels and Antioxidant Activity Assessment

RAW264.7 cells were seeded into 6-well plates (3 × 10^5^ cells/well) overnight. The cells were incubated with different concentrations (1, 5, 25, and 125 μg/mL) of ATR for 8 h, after which they were further stimulated for an additional 12 h with 10 ng/mL of LPS. The levels of cytokines were measured using a TNF-α, IL-6 ELISA kit (LIANKE, Hangzhou, China) in accordance with the protocol of the manufacturer. Following the manufacturer’s instructions, the superoxide dismutase (SOD), malondialdehyde (MDA), and glutathione (GSH) levels were quantified using a commercial kit (A001-3; A003-1; A006-2-1; Nanjingjiancheng, Nanjing, China). The TNF-α and IL-6 results were expressed in pg/mL; The SOD results were expressed in nmol/mg; the MDA results were expressed in nmol/L; and the GSH results were expressed in μmol/L.

#### 4.3.3. Reactive Oxygen Species Measurements

The intracellular ROS levels were measured using a ROS Assay Kit (Beyotime Biotechnology, Shanghai, China) and 2′,7′-dichlorofluorescein-diacetate (DCFH-DA), which is easily oxidized into fluorescent dichlorofluorescein (DCF) by intracellular ROS, its principal component, and therefore, the ROS levels were quantified. Briefly, the cells were seeded in 6-well plates as described above and exposed to various concentrations of ATR. Subsequently, the cells were evaluated for total ROS via flow cytometry.

#### 4.3.4. Western Blotting

HT29 or Caco2 cells were seeded into 6-well plates (3 × 10^5^ cells/well) and incubated overnight. The RAW264.7 cells were treated with LPS (10 μg/mL) for 4 h and then incubated with DMSO, ATR (25 μg/mL), and dexamethasone (Dex) (50 nM) for 24 h. HT29 and Caco2 cells were cultured in the supernatant of RAW264.7 cells for 12 h. The extraction of total proteins from HT29 and Caco2 cells was performed using lysis RIPA buffer (Beyotime Biotechnology, Shanghai, China). Then, the protein concentration was measured using a BCA protein assay kit (Beyotime Biotechnology, Shanghai, China). Forty micrograms of total protein was separated via 10% sodium dodecyl sulfate–polyacrylamide gel electrophoresis. Subsequently, the samples were transferred to a PVDF membrane for 1 h in a 100 V constant-pressure cold bath. The PVDF membrane was soaked in TBST (blocking solution) containing 5% skim milk powder for 30 min before being sealed on a shaking table at room temperature. The membrane and primary antibody were then incubated at 4 °C overnight. The PVDF membrane was submerged in a diluted HRP-labeled secondary antibody (1:5000) and incubated at 37 °C for 2 h on a shaker. Using an enhanced chemiluminescence (ECL) detector, immune-active proteins were identified. BioRad’s device assessed the signal strength of each protein band.

### 4.4. Analysis of Phosphospecific Protein Microarray

The LPS-induced RAW264.7 inflammatory cell model was used to extract protein from Full Moon lysate. The procedures of protein extraction, biotin labeling, chip closure, and chip hybridization were performed according to the standard operation flow of Full Moon, and the chip was scanned with a SureScan Dx Microarray Scanner. GenePix Pro 6.0 is capable of reading raw data from scanned chip images, as well as fluorescence signals and background. The mean, SD, and CV of the repeated correction signal values of each antibody were calculated twice, and the mean was taken as the signal value of each antibody for subsequent analysis. The phosphorylation level of a protein at the phosphorylation site was obtained by dividing the phosphorylation antibody signal value by the nonphosphorylation antibody signal value. The experimental group was compared with the control group, and the phosphorylation site modulation calculation was performed using the following formula:

$${\mathrm{Phosphorylation}}_{\mathrm{ratio}}=\frac{\mathrm{phosphoA}/\mathrm{unphosphoA}}{\mathrm{phosphoB}/\mathrm{unphosphoB}}$$

The signals of the phosphorylated and nonphosphorylated proteins from the experimental samples were indicated as phosphoA or phosphoB, and unphosphoA or unphosphoB, respectively. The fold-change threshold for the experimental group/control group was taken as 1.35 when the number of phosphorylated sites with intergroup modulation differences was controlled at approximately 10–30% of the total number of detected proteins. GO and KEGG pathway enrichment analyses and functional annotation analyses were performed on the differentially phosphorylated proteins between groups.

### 4.5. Establishment of the Ulcerative Colitis Model in Mice

Female Balb/c mice (18–20 g) were purchased from B&K Universal Group Limited. All mice were kept in specific pathogen-free (SPF) barrier conditions in a temperature-controlled room (22–24 °C) and were housed in standard mouse cages with ad libitum access to water and food for one week before the experimentation. All animal care and experimental protocols followed the regulations of the Shanghai University of Traditional Chinese Medicine Institutional Animal Care and Use Committee (IACUC).

All mice were housed in groups of five mice per cage and acclimatized for one week before being included in this study. Mice received 3% DSS (40 kDa; MP Biomedicals, Tokyo, Japan) supplemented in their drinking water for 7 d, followed by normal water. The control group of healthy mice received normal water exclusively. Then, 50 mg/kg of ATR or 150 mg/kg of 5-ASA was administered orally to mice on predetermined days. Changes in body weight were assessed daily during the 10 d experimental period. Feces were collected on a predetermined day for disease activity index (DAI) analysis and rated using the scoring criteria (Table S1). On the final day of the experiment, the mice were euthanized, and the entire colons were surgically removed. Colon lengths were measured, and then the colons were gently washed with physiological saline.

### 4.6. GC-TOF-MS Conditions

Gas chromatography was performed using an Agilent J & W Scientific DB-5MS capillary column (30 m × 250 µm i.d., 0.25 µm film thickness) with a constant flow of 1 mL/min of helium. Samples were injected in split mode at a 1:10 split ratio with the auto-sampler, and the injection temperature was set at 280 °C. The transfer line ion source temperature was set at 320 °C and 230 °C, respectively. The temperature programming followed an initial temperature of 50 °C for 0.5 min, with a rate of 15 °C/min up to 320 °C, and was held at 320 °C for 9 min. The mass spectrometry utilized a full scan method with a scan rate of 10 spec/s, electron energy of −70 V, and a solvent delay of 3 min.

### 4.7. Data Processing

The metabolomics data underwent quality control and batch correction using the MetaboAnalyst4.0 R package in R programming language. Unsupervised dimensionality reduction analysis (PCA) was utilized to identify characteristic metabolites, and a parallel pathway analysis of these characteristic metabolites was conducted.

### 4.8. Statistical Analysis

All experiments were repeated at least three times, and the data are presented as means ± SD. The differences between groups were determined using one-way ANOVA analysis, and the comparison between the two groups was analyzed with the *t*-test using GraphPad Prism 7.0 (GraphPad Software Inc., La Jolla, CA, USA). The differences were considered statistically significant when *p* < 0.05 and highly significant when *p* < 0.001.

## 5. Conclusions

**Figure 7:**
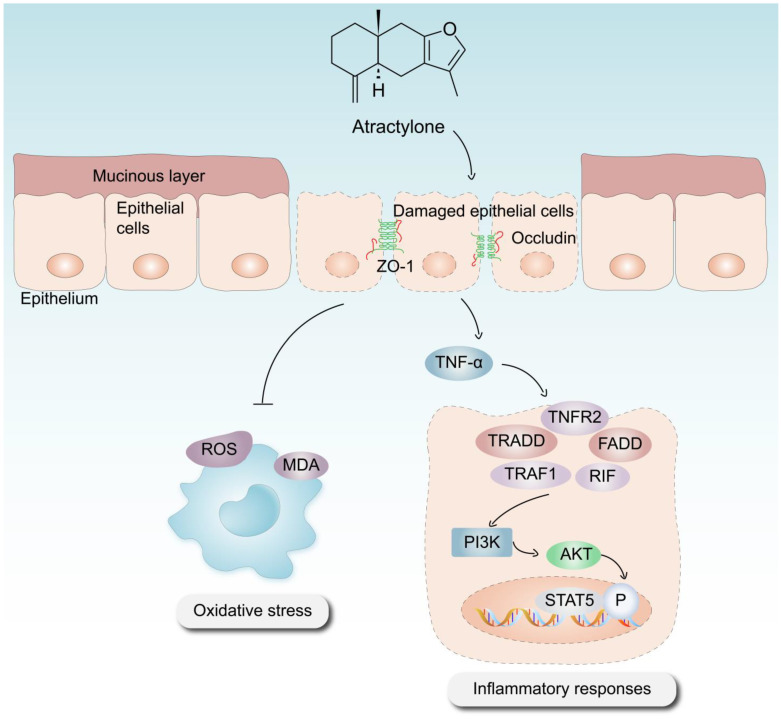
Mechanism via which ATR mitigates DSS-induced UC.

In conclusion, the significance of ATR in the treatment of UC has been substantiated using various methodologies, including in vitro and in vivo experimentation, as well as the use of phosphorylated protein chips. ATR primarily exerts its effects on colitis through anti-inflammatory, antioxidative stress, intestinal damage repair, and other mechanisms (Figure 7). Our results suggest that the use of AO treatment, especially the optimal concentration of ATR, confers therapeutic advantages in the context of colitis by prohibiting the expression of proinflammatory cytokines such as IL-6 and TNF-α. This observed prohibition is likely achieved by the downregulation of the PI3K–AKT signaling pathway, which serves to enhance the repair of intestinal epithelial barrier function. The metabolomics results reveal that ATR plays a crucial role in the regulation of amino acid metabolism, hence contributing to the mitigation of colonic injury in murine models of colitis. Future research is still needed to explore the interaction between the active component and the signaling pathway involved.

## Data Availability

Not applicable.

## Supplementary Materials

The following supporting information can be downloaded at https://www.mdpi.com/article/10.3390/molecules28217340/s1, Table S1: Disease activity index (DAI) scoring criteria; Figure S1: Enrichment analysis of over-representation analysis.
