# Supplementary material for: Atractylone in the Atractylodes macrocephala Rhizoma Essential Oil and Its Anti-Inflammatory Activity
Source: Molecules. 2023 Oct 30;28(21):7340. doi: 10.3390/molecules28217340 (PMC10648463; doi:10.3390/molecules28217340)
Supplement: Supplementary file 1 [file molecules-28-07340-s001.zip › molecules-2646656-SI.pdf]

Supplementary Materials

Atractylone in the *Atractylodes macrocephala Rhizoma* Essential Oil and Its Anti-Inflammatory Activity

Supplementary Tables

Supplementary Table S1. Disease Activity Index (DAI) scoring Criteria

Score	Weight loss (%)	Feces state	Occult blood test
0	0	Normal stool	Negative
1	1-5	Soft stool	Slight positive
2	6-10	Loose stool	Positive
3	11-15	Watery stool	Strong positive
4	>15	Diarrhea	Bloody stool with the naked eye

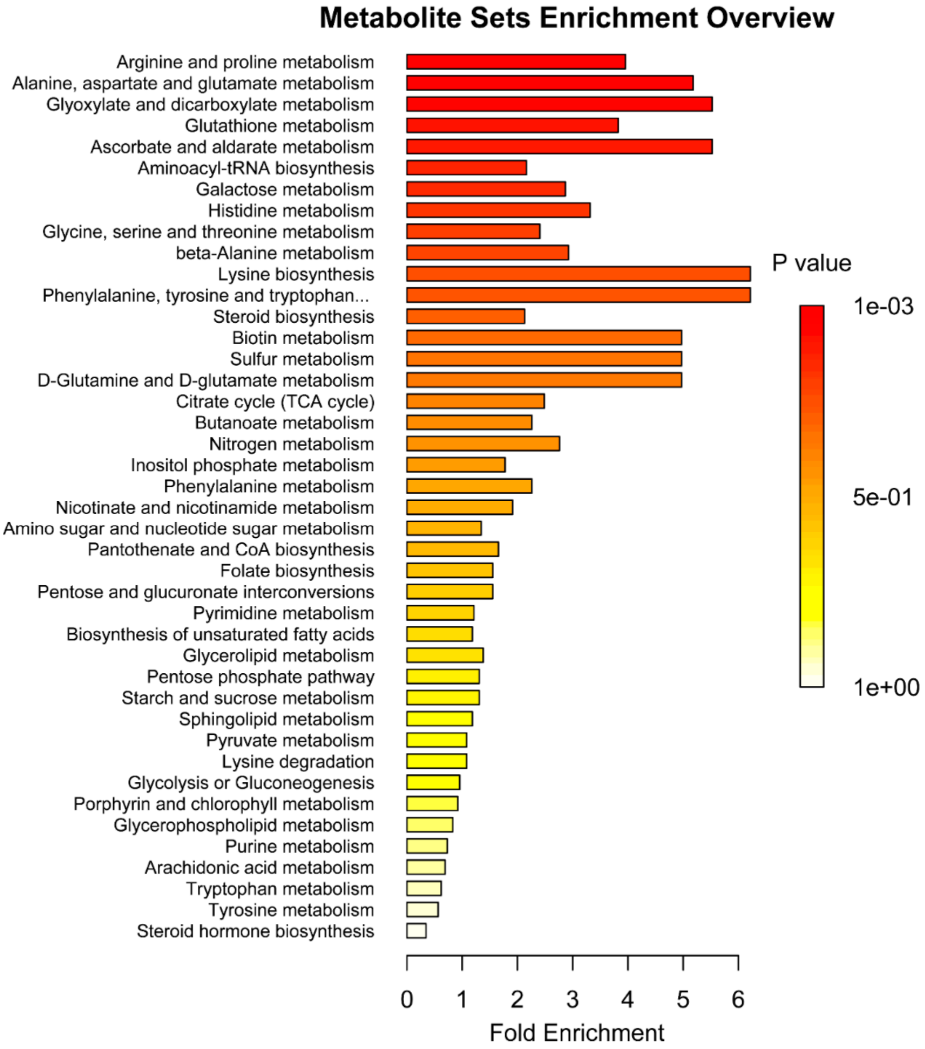

Figure S1. Enrichment analysis of over-representation analysis.
